# Co-amoxiclav-induced Stevens Johnson Syndrome in a child

**DOI:** 10.11604/pamj.2013.14.38.1408

**Published:** 2013-01-27

**Authors:** Neila Fathallah, Zayani Hanen, Raoudha Slim, Lobna Boussofara, Ghariani Najet, Kamel Bouraoui, Chaker Ben Salem

**Affiliations:** 1 Professor of Clinical Pharmacology, Department of Clinical Pharmacology, Faculty of Medicine of Sousse, Tunisia; 2 Emergency Health Technician, Intensive Care Department, Sahloul Hospital, Sousse, Tunisia; 3 Assistant professor of Dermatology, Farhat Hached University Hospital, Sousse, Tunisia

**Keywords:** Stevens Johnson syndrome, co-amoxiclav, child

## Abstract

Stevens-Johnson Syndrome is an uncommon life threatening disease generally induced by drugs. Antibiotics, mainly sulphonamides, are the most involved drugs in Stevens-Johnson Syndrome in children. Co-amoxiclav is a well tolerated antibiotic. It has never been reported to cause, lonely this syndrome in children. Herein, we report a co-amoxiclav-induced Stevens-Johnson Syndrome occurring in an 18-month-old child. The diagnosis of SJS is often challenging in children and other possible diseases should be ruled out. The etiology of this syndrome is not yet fully understood. It is thought to be mediated by an immunologic mechanism. Management involves early identification, withdrawal of the culprit drug and rapid initiation of supportive therapies.

## Introduction

Stevens-Johnson syndrome (SJS) is a mucocutaneous disease associated with significant morbidity and mortality. It is a relatively uncommon disorder in children and it is generally induced by medications [1]. Antibiotics, mainly sulphonamides, are the most involved drugs in SJS in children. Co-amoxiclav is a generally well tolerated antibiotic. It has never been reported to cause, lonely this syndrome in children. Herein, we report the first case of co-amoxiclav-induced SJS occurring in an 18-month-old child.

## Patient and observation

**Figure 1:**
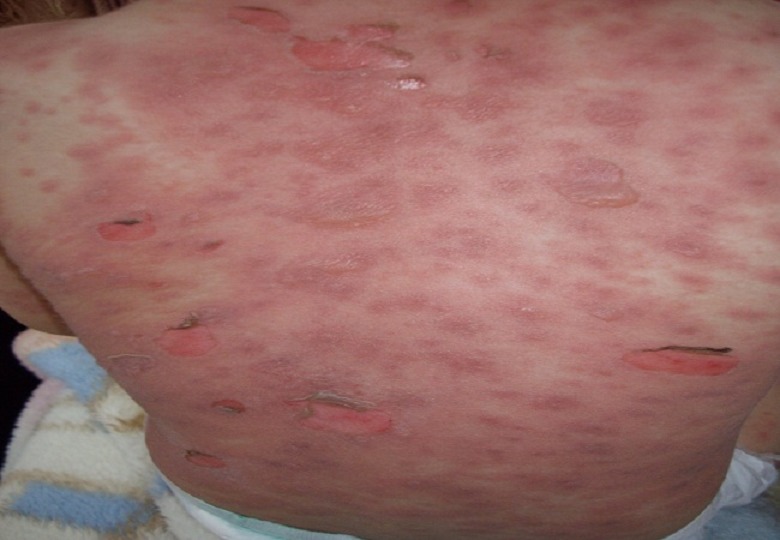
Erythematous rash with flaccid blisters affecting the trunk

An 18-month-old child was admitted to general pediatric department for accidental ingestion of caustic solution. He had no significant past medical history. Early esophagogastric endoscopy was performed showing severe esophagogastric necrosis. Parenteral nutrition was started and the child was treated by systemic corticosteroids (0.1 mg per kg daily) and omeprazole (10 mg daily). He was discharged home several days later with co-amoxiclav (50 mg per kg daily) on his medical prescription for productive cough. Three days later, he was readmitted to the pediatric department, with a chief complaint of fever and erythematous and bullous rash. He had no known drug allergies. Physical examination was remarkable for fever at 39°C, and a generalised erythematous rash with flaccid blisters filled with serous liquid involving large areas of the body surface (Figure 1). His eye, mouth, pharyngeal and genital mucosae were also affected by erosive lesions. Nikolsky's sign was positive. Epidermal detachment was observed over 5% of the body surface area (BSA).

Laboratory investigation showed the following: white blood cell count of 13.3 ×10^9^/L (normal range: 4-10 ×10^9^/L); there was no eosinophilia. Liver enzymes were within normal range: aspartate aminotransferase was 32 UI/L (normal range: 3-40 IU/L); alanine aminotransferase was 13 UI/L (normal range: 3-45 IU/L). Prothrombin time was 100%. Renal function tests were normal. C-reactive protein was 12 mg/dL. Both viral and bacterial infections including Epstein Barr Virus (EBV), Cytomegalovirus (CMV) and Mycoplasma pneumonia were negative. Blood cultures were also negative.

Co-amoxiclav-induced SJS was suspected. The drug was immediately withdrawn. The patient was started on intravenous corticosteroid and skin care. The outcome was favourable. The lesions improved progressively and the patient was discharged home with instructions to avoid co-amoxiclav.

## Discussion

SJS is a rare immune-mediated cutaneous reaction associated with significant morbidity and mortality especially in children. Two multicenter international case-control studies reported that less than 10% of cases occurred in children [2, 3]. Clinically, SJS in children shares the same characteristics with those in adults, it predominantly involves the skin and mucous membranes and it is characterized by an epidermal detachment below 10% of the BSA.

Drugs are considered the primary cause of SJS. Antimicrobials such as sulfonamides are among the most commonly implicated drugs in SJS occurring in children [4]. Co-amoxiclav is a generally well tolerated antimicrobial. Its most frequently reported adverse effects are gastrointestinal adverse reactions and hepatotoxicity [5]. It has been reported in few publications as etiology of SJS in adults [6]. To the best of our knowledge, co-amoxiclav has never been reported to cause SJS in children. The unique case found in the literature was of a 2 years and 2 months old girl that developed SJS and cholestatic hepatitis following treatment with co-amoxiclav and associated with EBV infection. EBV infection and co-amoxiclav consumption may be possible causes of SJS in the case reported by Maggio MC [7]. Infectious diseases such as human immunodeficiency virus, herpesvirus, Mycoplasma pneumonia, and hepatitis A virus are factors highly associated with SJS.

Our patient presented with SJS few days after treatment with co-amoxiclav which seemed to be the most probable cause of this dermatological condition. According to objective causality assessment using the Naranjo probability scale, co-amoxiclav-induced SJS was probable in our patient.

The diagnosis of SJS is often challenging in children and other possible diseases should be ruled out. Staphylococcal scalded skin syndrome, fixed bullous drug eruption, linear Ig A dermatosis, drug-induced pemphigus and bullous pemphigoid and acute generalised exanthematous pustulosis are differential diagnoses that should also be considered [8] Staphylococcal scalded skin syndrome is linked to a phage group 2 *Staphylococcus aureus* infection. Distinction from SJS is based on the absence of an antecedent macular rash (infrequently there is a scarlatiniform eruption) and absence of mucosal lesions and of internal organ involvement.

The etiology of SJS is not fully understood. Immunologic mechanisms, reactive drug metabolites, or interactions between these two have been reported in several reviews. Specific drug hypersensitivity induces a major histocompatibility class I-restricted drug presentation and leads to an expansion of cytotoxic T lymphocytes, and consequently an infiltration of skin lesions with cytotoxic T-lymphocytes and natural killer cells. Other findings suggest activation of the perforin/granzyme pathway as a cytotoxic mechanism in SJS. Recent findings suggest that granulysin probably is the key mediator for disseminated keratinocyte death in SJS [9].

Management of SJS involves early identification and withdrawal of the culprit drug, rapid initiation of supportive care by fluid and electrolyte replacement, acid-base and metabolic equilibrium regulation, serum protein and blood glucose control and topical skin management. Adjuvant treatments such as corticosteroids and immunosuppressants may also be used in severe cases of SJS [9].

## Conclusion

Clinicians should be conscious of the risk of co-amoxiclav-induced SJS in children in order to avoid a fatal outcome. Management involves early identification, withdrawal of the culprit drug and rapid initiation of supportive therapies.
